# Single-Molecule FISH Reveals Subcellular Localization of α-Amylase and Actin mRNAs in the Filamentous Fungus *Aspergillus oryzae*

**DOI:** 10.3389/fmicb.2020.578862

**Published:** 2020-09-22

**Authors:** Yujiro Higuchi, Kaoru Takegawa

**Affiliations:** Department of Bioscience and Biotechnology, Faculty of Agriculture, Kyushu University, Fukuoka, Japan

**Keywords:** single-molecule FISH, gene expression, mRNA, actin, *Aspergillus oryzae*, α-amylase

## Abstract

The machinery for mRNA localization is one of crucial molecular structures allowing cellular spatiotemporal organization of protein synthesis. Although the molecular mechanisms underlying mRNA localization have been thoroughly investigated in unicellular organisms, little is known about multicellular and multinuclear filamentous fungi. Here, we conducted single-molecule fluorescence *in situ* hybridization (smFISH) to first visualize the mRNA molecules of α-amylase, which are encoded by *amyB*, and which are thought to be abundantly secreted from the hyphal tips of the industrially important fungus *Aspergillus oryzae*. Consistent with previous biochemical studies, fluorescein amidite (FAM) fluorescence derived from *amyB* expression was observed in *A. oryzae* hyphae cultured in a minimal medium containing maltose instead of glucose as the sole carbon source. Moreover, after more than 1 h incubation with fresh maltose-containing medium, the fluorescence of *amyB* mRNAs was observed throughout the cells, suggesting α-amylase secretion potentially from each cell, instead of the hyphal tip only. Furthermore, in cultures with complete medium containing maltose, *amyB* mRNAs were excluded from the tip regions, where no nuclei exist. In contrast, mRNAs of actin, encoded by *actA*, were localized mainly to the tip, where actin proteins also preferentially reside. Collectively, our smFISH analyses revealed distinct localization patterns of α-amylase and actin mRNAs in *A. oryzae* hyphal cells.

## Introduction

The localization of mRNA is a fundamental molecular mechanism for regulating cellular and physiological processes (Buxbaum et al., 2015; Vera et al., 2016; Mofatteh and Bullock, 2017). The visualization of mRNAs has been conducted in a wide range of organisms, from bacteria to eukaryotes. Single-molecule fluorescence *in situ* hybridization (smFISH), which uses multiple fluorescent probes to enable visualization of a single mRNA molecule, is one of powerful methods for investigating mRNA localization. The technique has been used to investigate many organisms, including yeasts and neurons (Raj et al., 2008; Eliscovich et al., 2017; Tutucci et al., 2018). Such studies have intensely analyzed the molecular machinery underlying the subcellular localization of actin mRNAs and have revealed some of the functional and structural asymmetries in a variety of cell types (Buxbaum et al., 2015; Mofatteh and Bullock, 2017). However, in most cases, these investigations have been performed in unicellular models, and thus the information on mRNA localization in multicellular systems is limited.

In multicellular cells for studying the regulation of gene expression, the fruit fly *Drosophila* is known to be a model organism, in which morphogens are key factors to organize gene expression (Tabata and Takei, 2004; Bakker et al., 2020). However, little is known about how gene expression is regulated in other multicellular organisms, including filamentous fungi. Filamentous fungal cells are highly polarized and contain multiple nuclei in each cell, where generally the nucleus localized closest to the apex is at least 10 μm or farther from the tip, unless the machinery for cell division is perturbed (Morita et al., 2020). Therefore, there should be certain molecular mechanisms that orchestrate gene expression throughout each cell (Steinberg et al., 2017). Indeed, smFISH analysis applied to the G1 cyclin transcript of the multinucleated filamentous fungus *Ashbya gossypii*, which is closely related to the budding yeast *Saccharomyces cerevisiae*, revealed regulatory mechanisms of the cell cycle (Lee et al., 2013). In filamentous fungi, the regulation of cell cycle is distinct; for example, mitosis in *A. gossypii* is asynchronized, but it is synchronized in *Aspergillus* spp. (Gladfelter, 2006; Yasui et al., 2020). Although biochemical analysis to determine mRNA expression in total cell cultures have been widely performed, only a few cell biological investigations of mRNA localization have been reported in filamentous fungi.

The industrially important filamentous fungus *Aspergillus oryzae* produces abundant quantities of valuable secretory enzymes such as α-amylase and certain specific secondary metabolites (SMs) such as kojic acid (Machida et al., 2005; Kitamoto, 2015). Especially in *A. oryzae*, α-amylase mRNAs are highly expressed, and the molecular regulation of α-amylase mRNA has been well characterized (Gomi, 2019). α-Amylase randomly hydrolyzes the α-1,4 linkage of starch. It is encoded by 3 almost identical genes, *amyA*, *amyB*, and *amyC*, among which *amyB* is the most highly expressed in *A. oryzae* (Tada et al., 1989). The expression of α-amylase genes are repressed in the presence of glucose, which acts in a negative feedback mechanism. In addition, it is known that maltose is an inducing molecule for expression of not only the α-amylase genes, but also other related starch-degrading enzymes. On the other hand, regarding the protein levels of α-amylase, *in vivo* imaging has revealed brightly fluorescent enhanced green fluorescence protein (EGFP)-tagged AmyB in the apical vesicle cluster Spitzenkörper, suggesting that α-amylase is mainly secreted from the hyphal tip (Kimura et al., 2010). Moreover, some of the molecules of AmyB-EGFP were localized to septa, suggesting that there is a septum-directed secretion mechanism for α-amylase (Hayakawa et al., 2011). Furthermore, the actin cytoskeleton that is primarily localized at the hyphal tip is required for α-amylase secretion from the hyphal tip but not for α-amylase secretion from the septum (Hayakawa et al., 2011). Although this molecular mechanism for α-amylase secretion has been investigated, where and how α-amylase mRNAs are transcribed and translated in *A. oryzae* multicellular and multinuclear hyphae are not at all understood.

In this study, we performed smFISH analysis to visualize the subcellular localization of α-amylase mRNAs in *A. oryzae*. As expected, the expression of α-amylase mRNAs was induced in the presence of maltose as the sole carbon source, but not glucose. Importantly, maltose induced expression of α-amylase mRNAs throughout the cells, which suggest septum-directed and also intercalary secretion of α-amylase. Although it is well known that most α-amylase molecules are secreted through the hyphal tip and observed at the Spitzenkörper before secretion, α-amylase mRNAs are excluded from the hyphal tip, where actin mRNAs are preferentially localized. Collectively, these results suggest that distinct localization machinery for mRNAs exists in *A. oryzae* hyphal cells.

## Materials and Methods

### Strain and Cultures

*Aspergillus oryzae* wild-type strain RIB40 was used in this study. The strain RIB40 was grown on a plate of potato dextrose (PD; Nissui) for several days until a sufficient amount of conidia was formed. As a minimal medium, Czapek-Dox (CD; 0.3% NaNO_3_, 0.2% KCl, 0.1% KH_2_PO_4_, 0.05% MgSO_4_⋅7H_2_O, 0.002% FeSO_4_⋅7H_2_O and 2% glucose, pH 5.5) was used. To prepare maltose-containing CD medium (CDmal), the glucose in CD was replaced with maltose. As complete media, GPY or MPY (2% glucose or maltose, respectively, 1% polypeptone, 0.5% yeast extract, 0.5% KH_2_PO_4_, and 0.05% MgSO_4_⋅7H_2_O) were used. The liquid media described here were used for microscopy and were sterilized by filtering through 0.45 μm filters.

### Single-Molecule Fluorescence *in situ* Hybridization (smFISH)

Approximately 10^2^ to 10^3^ conidia of the strain RIB40 were inoculated into 100 μL of respective liquid media placed into poly-lysine coated glass bottom dishes (Matsunami) and incubated at 30°C for 12 h in complete medium or for 20 h in minimal medium. Single-molecule fluorescence *in situ* hybridization (smFISH) analysis was performed essentially according to the manufacturer’s instructions with some modifications based on previous literature (LGC Biosearch Technologies; König et al., 2009; Tutucci et al., 2018). Cultured cells were fixed with 100 μL of 7.4% formaldehyde in PBS (0.8% NaCl, 0.2% KCl, 0.12% Na_2_HPO_4_, 0.2% KH_2_PO_4_) at room temperature (RT) for 1 h. After undergoing 2 rinses with 100 μL of fixation buffer (1.2 M sorbitol, 0.1 M K_2_HPO_4_, pH 7.5), the cultures were permeabilized with 100 μL of 70% ethanol at −20°C overnight. The cells were then dissolved in 100 μL of hybridization buffer containing 10% deionized formamide with or without the addition of 1 μL of 12.5 μM of the probe (final concentration of 125 nM) overnight. The cells were then protected from the light for the procedures that follow. After replacement of the hybridization buffer with 100 μL of buffer A (LGC Biosearch Technologies), containing 10% of deionized formamide, the cells were incubated with 100 μL of buffer A at 30°C for 30 min. To stain the nuclei, the cells were incubated with 100 μL of buffer A containing 5 ng/mL DAPI in DMSO (Molecular Probes) at 30°C for 30 min. Buffer A was then replaced with 100 μL of buffer B (LGC Biosearch Technologies) and incubated at RT for 3 min. The cells were then mounted in 50 μL of Vectashield Antifade Mounting Medium (Vector Laboratories) to minimize fluorescence bleaching and subsequently observed under fluorescence microscope.

The smFISH probes (LGC Biosearch Technologies) were used according to the manufacturer’s instructions. The smFISH probe for *amyB* consisted of mixtures of 18–22 nt from 47 regions in 1,497 b, each region was linked to fluorescein amidite (FAM; excitation/emission, 495/520 nm). The smFISH probe for *actA* consisted of mixtures of 18 to 22 nt from 45 regions in 1,128 b, each region was linked to CAL Fluor Red 610 (excitation/emission, 590/610 nm). The probe sequence data are summarized in Supplementary Figures S1, S2.

### Fluorescence Microscopy

We used an ECLIPSE Ti2-A inverted microscope (Nikon) equipped with a CFI Plan Apo Lambda 100 × objective lens (1.45 numerical aperture), a DS-Qi2 digital camera, an LED-DA/FI/TX-A triple band filter (Semrock: Exciter, FF01-378/474/575; Emission, FF01-432/523/702; Dichroic mirror, FF409/493/596-Di02), an LED light source X-LED1 and differential interference contrast (DIC) to observe the FAM, CAL Fluor Red 610 and DAPI fluorescent signals and hyphal morphology of *A. oryzae* cells. Image data were acquired and merged by NIS Elements AR software (Nikon). Line scan analysis was carried out with the use of the intensity profile function in the NIS Elements AR software. The average fluorescence intensity was measured and corrected for the background intensity adjacent to the cell. Apical and subapical regions were categorized as 0–20 and 20–40 μm hyphal areas away from the tip, respectively, and basal regions were categorized as 20 μm hyphal regions from the germinated conidia. Microscopy for each experimental condition was performed independently at least three times, and representative images are shown.

### Statistical Analysis

Statistical analysis was performed by using Tukey–Kramer test for Figures 2C, 4 (statistically significant difference at *P* < 0.05) and Student’s *t*-test for Figure 6D.

## Results

### Subcellular Localization of α-Amylase mRNAs Cultured in Minimal Media

To visualize α-amylase mRNAs, we designed an smFISH probe based on the *amyB* sequence that encodes α-amylase. The *amyB* probe consisted of 47 regions of 18–22 nt from 1,497 b of the *amyB* sequence, and each region was linked to a FAM molecule, resulting in sufficient fluorescence for visualization of the *amyB* mRNAs (Supplementary Figure S1). The *A. oryzae* genome harbors 3 α-amylase genes, *amyA*, *amyB*, and *amyC*, the sequences of which are almost identical (Machida et al., 2005). Thus, the *amyB* probe probably cannot distinguish these mRNAs, and for simplicity, we used the term “*amyB*” or “α-amylase mRNAs” in this manuscript. Generally, fluorescence microscopic analysis of fungal cells is performed for cultures in minimal medium, because complete medium leads to higher background fluorescence than minimal medium. In addition, *amyB* is known to be abundantly expressed when *A. oryzae* cells are cultured in medium containing maltose as the carbon source (Tada et al., 1989).

**FIGURE 1:**
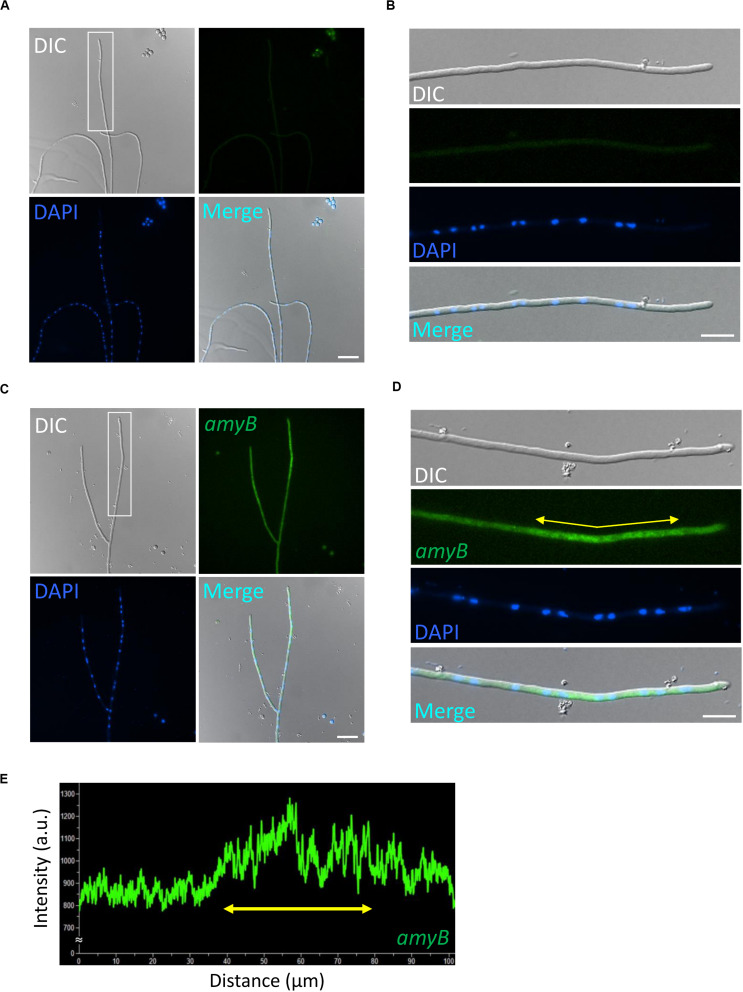
Localization of *amyB* mRNAs in *A. oryzae* hyphae cultured in CDmal medium. smFISH was performed without **(A,B)** or with **(C,D)** the *amyB* probe. The images in **(B,D)** are magnifications of the regions within the white rectangles in **(A,C)**, respectively. **(E)** Line scan analysis of *amyB* fluorescent signals was conducted through the hypha shown in **(D)**. Yellow arrows in **(D,E)** depict corresponding regions. Note that the subapical areas show more intense fluorescent signals than the tip region. Scale bars, 20 μm **(A,C)** and 10 μm **(B,D)**.

Thus, we first performed smFISH on *A. oryzae* cultures grown in CDmal medium with or without the *amyB* probe (Figure 1). In the negative control culture processed without the *amyB* probe, the hyphal cells emitted minimal fluorescence associated with *amyB*, with the exception of the conidia; thus, we reasoned that conidial fluorescence did not represent positive signals from *amyB* mRNAs (Figures 1A,B). At the same time, we successfully performed DAPI staining to visualize the nuclei and observed DAPI fluorescence in these smFISH samples, indicating that the permeabilization procedure was effective. By contrast, in cultures processed with the *amyB* probe, we observed that the hyphal cells emitted FAM fluorescence (Figure 1C). In enlarged images, *amyB* fluorescence was observed not at the apical but at the subapical region (Figures 1D,E). Since *amyB*-encoded α-amylase is thought to be mainly secreted from the hyphal tip (Kimura et al., 2010), our result suggests that *amyB* mRNAs and its proteins are localized to regions that are somehow distinct from each other.

**FIGURE 2:**
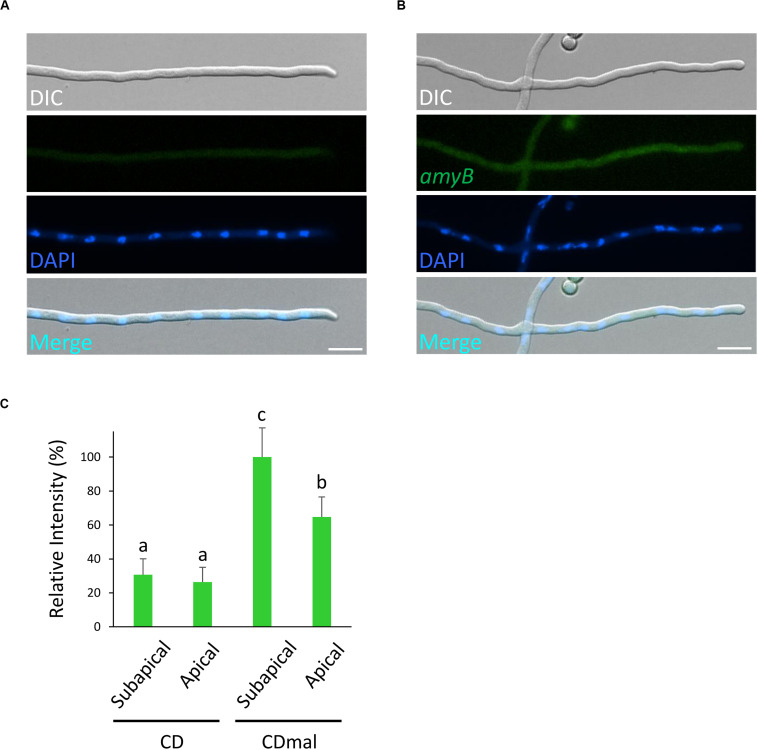
Localization of *amyB* mRNAs in *A. oryzae* hyphae cultured in CD medium that contains glucose as the sole carbon source. smFISH was performed without **(A)** or with **(B)** the *amyB* probe. Scale bars, 10 μm. **(C)** Relative fluorescence intensity of *amyB* mRNAs at the apical and subapical regions of cultured cells in CD and CDmal media. Bars show mean with standard deviation. Significant difference at *P* < 0.05 among each lowercase (Tukey–Kramer test, *n* = 10).

Next, to evaluate the fluorescence of *amyB* mRNAs, we performed smFISH on cultures in CD, which contained glucose as the carbon source, with or without the *amyB* probe (Figures 2A,B). Since *amyB* is known not to be expressed in cultures with media containing glucose (Tada et al., 1989), we did not expect to see fluorescent signals from the glucose-containing cultures. Indeed, we observed minimal fluorescence, even with the addition of the *amyB* probe from the glucose-containing cultures. We further performed quantitative fluorescence intensity analysis and found that *amyB* mRNAs cultured in CDmal medium were significantly more expressed than those cultured in CD medium (Figure 2C). Moreover, in CDmal cultures, the quantitative intensity analysis confirmed that *amyB* mRNAs were localized significantly more at subapical regions than at apical regions. Altogether, these results suggest that our *amyB* probe smFISH analysis effectively identifies *amyB* mRNAs in *A. oryzae* hyphae.

### Induced Expression of α-Amylase mRNAs Cultured With Maltose as the Sole Carbon Source

**FIGURE 3:**
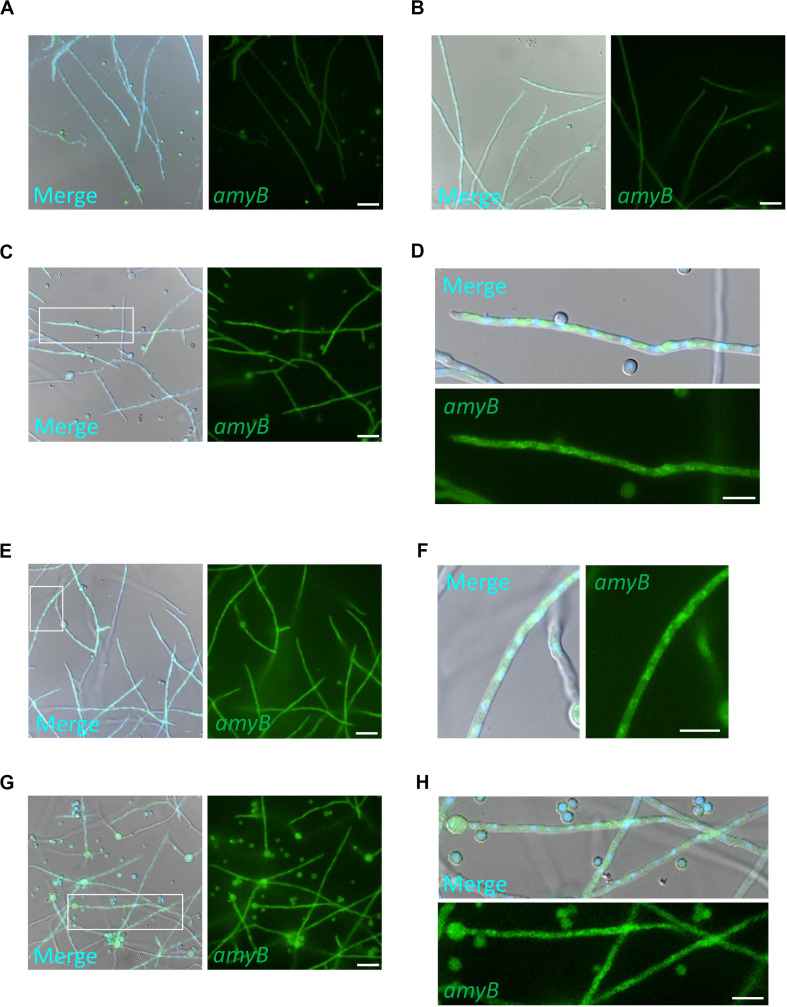
Expression of *amyB* mRNA by shifting media from CD to CDmal. smFISH was performed with the *amyB* probe in cultures of CD before shifting **(A)** and after shifting to CDmal, at 30 min **(B)**, 60 min **(C,D)**, and 90 min **(E–H)**. Note that *amyB* fluorescent signals were observed through the hyphae **(E,G)**. The images in **(D,F,H)** are magnifications of the regions within the white rectangles in **(C,E,G)**, respectively. These enlarged images show *amyB* fluorescent signals in the apical **(D)**, intermediate **(F)** and basal regions **(H)**. Scale bars, 20 μm **(A–C,E,G)** and 10 μm **(D,F,H)**.

**FIGURE 4:**
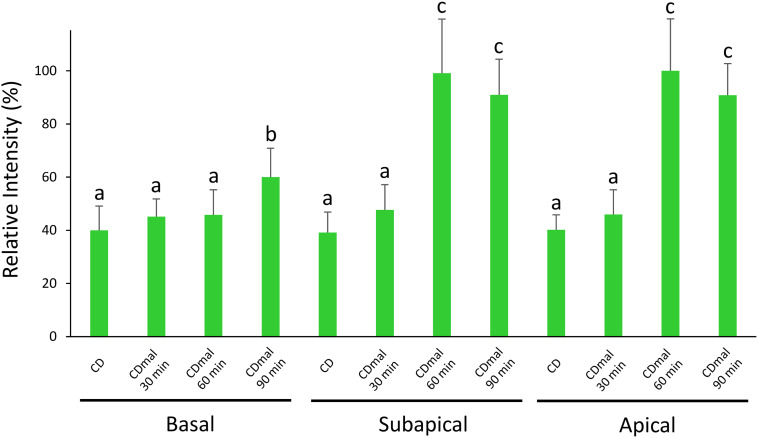
Relative fluorescence intensity of *amyB* mRNAs at the apical, subapical and basal regions of cultured cells in CD before shifting and after shifting to CDmal, at 30, 60, and 90 min. Bars show mean with standard deviation. Significant difference at *P* < 0.05 among each lowercase (Tukey–Kramer test, *n* = 10).

To further characterize the expression profiles of *amyB* mRNAs, we performed medium-shifting from glucose to maltose as the sole carbon source (Figure 3). The medium shifting from glucose-containing medium (Figure 3A), to maltose-containing medium induced *amyB* fluorescence (Figures 3B–H). Culture in maltose-containing medium for 30 min did not fully induce *amyB* fluorescence, whereas culture for longer than 60 min resulted in *amyB* fluorescence from each hyphal cell (Figures 3B,C,E,G). These time-course results of *amyB* induction were consistent with a previous biochemical study (Suzuki et al., 2015). Instead of a biochemical analysis to determine the mean response of the entire cell culture, the cell biological analysis by smFISH in this study revealed that *amyB* expression occurred not only in the apical regions but also in the intermediate and even basal regions of the hyphae (Figures 3D,F,H). We also performed negative control experiments processed without the *amyB* probe, and confirmed that minimal fluorescence associated with *amyB* appeared (Supplementary Figure S3). Our quantitative fluorescence intensity analysis clearly showed that *amyB* mRNAs were significantly more expressed from 60 min after shifting to the maltose-containing medium (Figure 4). Moreover, we found that newly synthesized *amyB* mRNAs were also localized to the apical regions and that significant increase of *amyB* mRNAs fluorescence at basal regions was observed at 90 min after the medium shifting. Collectively, these results of the medium-shifting investigations strongly suggest that *amyB* fluorescence indeed reflects the subcellular localization of *amyB* mRNAs.

### α-Amylase mRNAs Are Excluded From the Hyphal Tip Region

**FIGURE 5:**
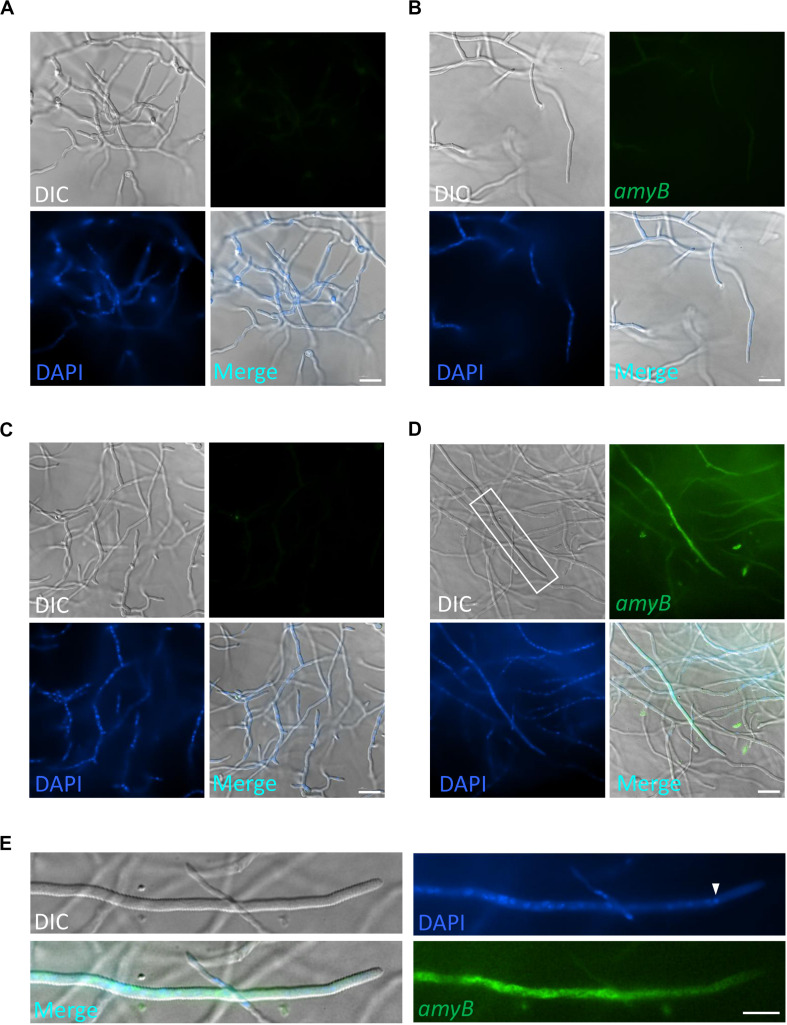
Localization of *amyB* mRNA in *A. oryzae* hyphae cultured in complete media. *A. oryzae* hyphae were grown in GPY **(A,B)** or MPY **(C,D)** media. smFISH was performed without **(A,C)** or with **(B,D)** the *amyB* probe. The images in **(E)** are magnifications of the region within the white rectangle in **(D)**. The white arrowhead indicates the nucleus located closed to the apex. Note that the *amyB* fluorescent signals are not seen in the tip region. Scale bars, 20 μm **(A–D)** and 10 μm **(E)**.

Our smFISH analyses hitherto used minimal media mainly to compare the majority of previous microscopy results. Here, we further investigated cells cultured in complete media, which are relevant to industrial needs, because for smFISH procedures, the complete media are replaced with a buffer that does not emit background fluorescence. First, we performed smFISH on cultures in GPY containing glucose as the carbon source and found minimal *amyB* fluorescence similar to cultures in CD (Figures 5A,B). Next, we performed smFISH on cultures in MPY containing maltose as the carbon source and found that *amyB* fluorescent signals were observed from the hyphal cells, in contrast to the negative control without the *amyB* probe (Figures 5C,D). We also found that *amyB* fluorescent signals were observed from the subapical regions but not from the apical regions (Figure 5E), similar to the results observed in CDmal cultures (Figure 1D). In addition, nuclei were not observed in the apical regions, which might account for the absence of *amyB* fluorescent signals from the apical regions.

**FIGURE 6:**
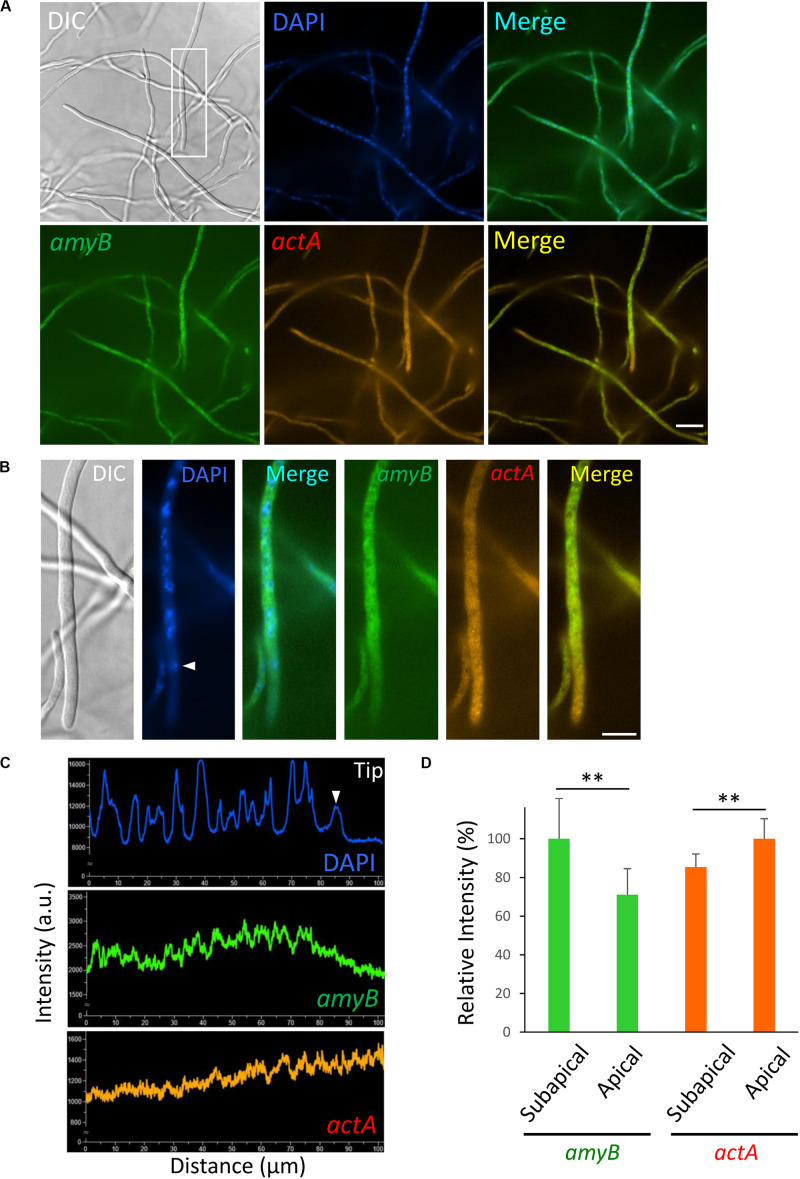
Localization of *amyB* and *actA* mRNAs in *A. oryzae* hyphae cultured in complete medium containing maltose. **(A)** smFISH was performed with *amyB* and *actA* probes added to an overnight culture in MPY. **(B)** Each image is a magnification of the region within the white rectangle in **(A)**. The white arrowhead indicates the nucleus located closed to the apex. **(C)** Line scan analyses of DAPI, *amyB* and *actA* mRNAs fluorescent signals were conducted through the hypha of **(B)**. The white arrowhead indicates the signal of the nucleus located closed to the apex. Note that the fluorescent signals of *amyB* mRNAs are not seen in the apical region, whereas those of *actA* mRNAs appeared more intense in the apical region. **(D)** Relative fluorescence intensity of *amyB* and *actA* mRNAs at apical and subapical regions. Bars show mean with standard deviation. **Statistically significant difference at *P* < 0.01 (Student’s *t*-test, *n* = 10). Scale bars, 20 μm **(A)** and 10 μm **(B)**.

### α-Amylase and Actin mRNAs Exhibit Distinct Subcellular Localization

Since we observed that localization of *amyB* mRNAs appeared excluded from the apical regions, we next investigated another mRNA molecule. The localization of actin mRNA has been extensively analyzed in a variety of eukaryotic organisms (Buxbaum et al., 2015; Mofatteh and Bullock, 2017); we thus investigated the localization of actin mRNA in *A. oryzae* hyphae. The *actA* gene encodes actin protein in *A. oryzae*. We designed an *actA* probe for smFISH, which consisted of 45 regions of 18–22 nt from 1,128 b of the *actA* sequence. Each region was linked to the CAL Fluor Red 610 fluorescence-emitting molecule (Supplementary Figure S2).

We performed smFISH by hybridizing both the *amyB* and *actA* probes with cultures of *A. oryzae* in MPY and found that *amyB* fluorescent signals were not seen from the tip regions of the hyphae, suggesting that the addition of the *actA* probe did not perturb the localization of *amyB* mRNAs (Figure 6A). In contrast, *actA* fluorescent signals appeared patchy and were mainly from the apical regions (Figures 6A,B). Line scan analyses clearly demonstrated that the *amyB* fluorescent signals were barely seen from the apical nucleus to the tip, whereas those of *actA* appeared even stronger from the apical regions (Figure 6C and Supplementary Figure S4). Further quantitative fluorescence intensity analysis confirmed that *amyB* mRNAs were localized significantly more at subapical regions than at apical regions, but in contrast *actA* mRNAs were localized significantly more at apical regions than at subapical regions (Figure 6D).

## Discussion

mRNA localization has been investigated in various cell types to understand how protein synthesis is spatially organized (Buxbaum et al., 2015). smFISH is one of the powerful cell biological tools that are used to analyze the subcellular localization of each mRNA molecule (Raj et al., 2008). The localization of mRNA in fungal cells by smFISH has been thoroughly investigated in yeast (Tutucci et al., 2018). By contrast, mRNA localization has not been comprehensively investigated in filamentous fungi, and only some studies in *A. gossypii* and the dimorphic fungus *Ustilago maydis* have used FISH analysis (König et al., 2009; Lee et al., 2013). Moreover, although cell biological expression analysis using the GFP reporter system for promoters of secretory proteins has been conducted in the industrial filamentous fungus *Aspergillus niger* (Vinck et al., 2011; Tegelaar et al., 2020), smFISH has not been used to directly visualize the mRNA molecules of secretory proteins in filamentous fungal cells. In this study, we applied the smFISH technique to *A. oryzae* cells and successfully obtained information on the subcellular localization of *amyB* and *actA* mRNAs, which encode the α-amylase and actin proteins, respectively.

**FIGURE 7:**
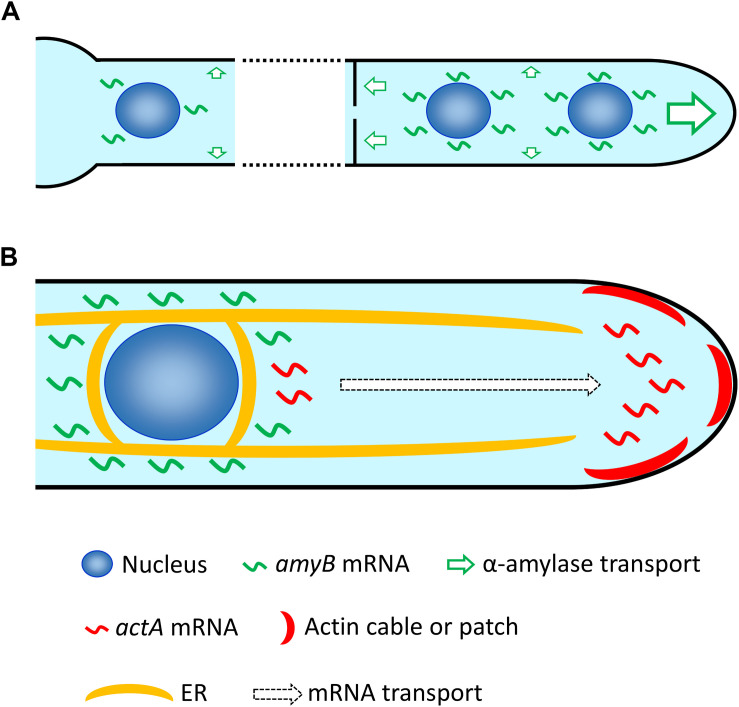
Hypothetical model of mRNA localization in *A. oryzae* hyphae. **(A)** After induction with maltose, *amyB* mRNAs are highly expressed at each nucleus through the hypha, which might result in not only primary apical secretion but also secretion through the septum, and even intercalary secretion of α-amylase. Note that for simplicity only a few nuclei in each cell are depicted, but generally *A. oryzae* hyphae contain multiple nuclei in every cell. **(B)** *amyB* mRNAs are restricted to locations near each nucleus and excluded from the tip region, and might be targeted to the ER for translation. In contrast, *actA* mRNAs are mainly localized to the apical region, where actin cables and patches preferentially reside. Since the nucleus closest to the apex normally localizes approximately more than 10 μm from the tip, *actA* mRNAs are potentially transported to the tip region after their transcription in the nucleus.

The expression profiles of *amyB* mRNAs revealed by smFISH were in strong accordance with two important attributes that were previously characterized biochemically (Suzuki et al., 2015; Gomi, 2019). First, maltose, but not glucose, is the key carbon source for *amyB* mRNA expression. Second, a sufficiently strong fluorescent signal from *amyB* mRNA expression occurs at about 1 h, or at least longer than 30 min after shifting from glucose-containing to maltose-containing culture medium. Importantly, our cell biological smFISH analyses revealed that *amyB* mRNA expression induced by the presence of maltose occurs throughout the hyphal cells, but appears not restricted at the apical regions from which the α-amylase protein is mainly secreted. Given that *de novo*-synthesized *amyB* mRNAs are locally translated, our observation raises the possibility that, in addition to α-amylase secretion mainly at the tip and supportively from the septum, intercalary secretion of α-amylase might also occur (Figure 7A). Indeed, in *A. oryzae*, some of soluble *N*-ethylmaleimide-sensitive factor attachment protein receptor (SNARE) proteins, which are required for protein secretion, are localized to the plasma membrane throughout the cells (Kuratsu et al., 2007). In addition, organelles of the secretory pathway, the endoplasmic reticulum (ER) and the Golgi apparatus, are also distributed throughout the cells (Kuratsu et al., 2007). However, the existence of intercalary secretion has not been clearly elucidated in filamentous fungi (Read, 2011), and thus, further cell biological investigations of secretary molecules that might also be dependent on cell culture conditions would reveal the underlying molecular mechanism of intercalary secretion.

We also revealed that the subcellular localizations of *amyB* and *actA* mRNAs are distinct, particularly at the hyphal tip region: *amyB* mRNAs appeared excluded from the tip, whereas *actA* mRNAs were mainly seen near the tip (Figure 7B). Since *amyB* mRNAs were not observed at the tip regions of hyphae cultured in both minimal and complete media, they may be immediately targeted to the ER for translation before being released to diffuse through the cytoplasm. Indeed, in *A. oryzae*, the ER is localized throughout the hyphal cells and normally surrounds the nuclei (Kuratsu et al., 2007; Togo et al., 2017; Morita et al., 2020). Thus, such preferential localization of *amyB* mRNAs might result in the efficient translation of large amounts of α-amylase for secretion. In contrast to *amyB* mRNAs, after transcription at the nuclei, *actA* mRNAs appeared to be mainly localized to the hyphal tip regions, where actin cables and patches have been observed (Higuchi et al., 2009; Hayakawa et al., 2011). Although the localization of actin proteins have been widely investigated in filamentous fungi (Araujo-Bazán et al., 2008; Upadhyay and Shaw, 2008; Berepiki et al., 2010; Delgado-Alvarez et al., 2010; Berepiki et al., 2011), to the best of our knowledge, ours is the first study and report of the visualization of actin mRNAs in these organisms.

In general, in the apical cells of *A. oryzae*, the nucleus localized closest to the apex is at least 10 μm or farther from the tip (Morita et al., 2020). Such apical organization of the cell is also seen in *Aspergillus nidulans*, and it is known that the bZIP transcription factor FlbB is transported between the tip and apex nucleus to become competent (Herrero-Garcia et al., 2015; Momany, 2015). Moreover, in *U. maydis* hyphae, there is such a distance between the tip and the nucleus where early endosomes transport possible signaling molecules and certain transcripts, such as septin mRNAs, with ribosomes to be locally translated at the tip (Baumann et al., 2014; Bielska et al., 2014; Higuchi et al., 2014; Higuchi and Steinberg, 2015). Although neither the mRNAs of secretory proteins nor the cytoskeleton has been investigated in filamentous fungi, mRNA transport mechanisms, including the mechanism for transporting *actA* mRNA, might also exist in *A. oryzae* hyphal cells. However, since smFISH can be used for fixed cells only, a live-imaging system for mRNAs is required to investigate the mRNA dynamics in *A. oryzae*, such as the MS2-MCP system, which has been used effectively for yeast cells (Tutucci et al., 2018).

Recently, expansion microscopy (ExM) has been successfully applied to fungal cells to visualize details of subcellular structures at a resolution of around 30 nm (Götz et al., 2020). Although similar to smFISH, ExM is also limited to available fluorophores and to fixed cells (Tillberg et al., 2016), physical expansion allows the sample to be observed at super-resolutions with conventional microscopy settings. Since complete digestion of the cell wall must be performed for ExM, its application to mature hyphal cells remains challenging because of their rigid cell wall structures. However, if both ExM and smFISH could be concomitantly applied, further details of mRNA localization could be elucidated; for example, membrane contact sites and membraneless organelles-associated mRNA molecules that are not well understood in filamentous fungi could be investigated (Lee et al., 2020). Moreover, mRNA localization in industrially relevant cultures with *A. oryzae*, such as solid-state fermentation, which are currently still challenging for cell biological approaches, could be investigated for further understanding of unidentified fungal cell biological aspects and potential industrial applications.

An understanding of mRNA localization in filamentous fungi confers various scientific benefits. For instance, the molecular mechanisms involved in the spatiotemporal regulation of mRNA expression in multinucleated and multicellular filamentous fungi remain unsolved and are fundamental to biological processes. Since there are plenty of cell biological researches conducted by using GFP fusion proteins, smFISH analysis with a *gfp* probe would provide comprehensive localization information between the corresponding protein and mRNA molecules. Additionally, in the context of biotechnology, the filamentous fungi produce a variety of valuable enzymes and bioactive SMs (Keller, 2015). SMs, especially, require several processing steps involving multiple enzymes for biosynthesis. The elucidation of not only enzymatic properties but also elucidation of the regulation of the times and locations of enzyme expression in filamentous fungal cells should provide beneficial information for further improving valuable material production.

## Data Availability Statement

The raw data supporting the conclusions of this article will be made available by the authors, without undue reservation.

## Author Contributions

YH performed the experiments, analyzed the data, and devised the project. YH and KT wrote the manuscript. Both authors contributed to the article and approved the submitted version.

## Conflict of Interest

The authors declare that the research was conducted in the absence of any commercial or financial relationships that could be construed as a potential conflict of interest.
