# Supplementary material for: Single-Molecule FISH Reveals Subcellular Localization of α-Amylase and Actin mRNAs in the Filamentous Fungus Aspergillus oryzae
Source: Front Microbiol. 2020 Sep 22;11:578862. doi: 10.3389/fmicb.2020.578862 (PMC7536267; doi:10.3389/fmicb.2020.578862)
Supplement: Supplementary file 1 [file Data_Sheet_1.pdf]

Supplementary Material

Single-molecule FISH reveals subcellular localization of α-amylase and actin mRNAs in the filamentous fungus *Aspergillus oryzae*

Yujiro Higuchi*, Kaoru Takegawa

Department of Bioscience and Biotechnology, Faculty of Agriculture, Kyushu University, 744 Motooka, Fukuoka 819-0395, Japan

*Corresponding author. Tel/Fax: +81 92 802 4734, E-mail address: y.higuchi@agr.kyushu-u.ac.jp

Word count: Abstract, 208; Main, 4390.

Number of figures: Main, 7; Supplementary, 4.

```
atgatggtcgcgtggtggtctctatttctgtacggccttcaggtcgcggcacctgctttg
         cgcaccaccagagataaaga
         Probe # 1

gctgcaacgcctgcggactggcgatcgcaatccatttatttccttctcacggatcgattt
                  accgctagcgttaggtaaat       gagtgcctagctaaa
                  Probe # 2                  Probe # 3

gcaaggacggatgggtcgacgactgcgacttgtaatactgcggatcagaaatactgtggt
cgttc                 gacgctgaacattatgacgc     ctttatgacacca
                      Probe # 4                Probe # 5

ggaacatggcagggcatcatcgacaagttggactatatccagggaatgggcttcacagcc
ccttgta                gttcaacctgatataggtcc       gaagtgtcgg
                       Probe # 6                  Probe # 7

atctggatcaccccgttacagcccagctgccccagaccaccgcatatggagatgcctac
tagacctagt                                 gtatacctctacggatg
                                           Probe # 8
catggctactggcagcaggatatatactctctgaacgaaaactacggcactgcagatgac
gta  gatgaccgtcgtcctatata gagagacttgcttttgatgc gtgacgtctactg
     Probe # 9            Probe # 10           Probe # 11

ttgaaggcgctctcttcggcccttcatgagagggggatgtatcttatggtcgatgtggtt
aacttcc  gagagaagccgggaagtact    ccctacatagaataccagct
         Probe # 12              Probe # 13

gctaaccatatgggctatgatggagcgggtagctcagtcgattacagtgtgtttaaaccg
 gattggtatacccgatacta      ccatcgagtcagctaatgtc        ttggc
 Probe # 14                Probe # 15                  Probe

ttcagttcccaagactacttccacccgttctgtttcattcaaaactatgaagatcagact
aagtcaagggttctg tgaaggtgggcaagacaaag        atacttctagtctga
 # 16           Probe # 17                  Probe # 18

caggttgaggattgctggctaggagataacactgtctccttgcctgatctcgataccacc
gtcca             gatcctctattgtgacagag    gactagagctatggtgg
                  Probe # 19              Probe # 20
aaggatgtggtcaagaatgaatggtacgactgggtgggatcattggtatcgaactactcc
ttc   caccagttcttacttaccat       caccctagtaaccatagctt
      Probe # 21                 Probe # 22

attgacggcctccgtatcgacacagtaaaacacgtccagaaggacttctggcccgggtac
        ggaggcatagctgtgtcatt  gtgcaggtcttcctgaagac
        Probe # 23            Probe # 24

aacaaagccgcaggcgtgtactgtatcggcgaggtgctcgacggtgatccggcctacact
                                                      atgtga
                                                      Probe
```

```
tgtccctaccagaacgtcatggacggcgtactgaactatcccatttactatccactcctc
acagggatggtctt          ccgcatgacttgatagggta atgataggtgaggag
# 25                    Probe # 26           Probe # 27

aacgccttcaagtcaacctccggcagcatggacgacctctacaacatgatcaacaccgtc
ttgcg aagttcagttggaggccgtc tacctgctggagatgttgta tagttgtggcag
      Probe # 28           Probe # 29           Probe # 30
aaatccgactgtccagactcaacactcctgggcacattcgtcgagaaccacgacaaccca
tttaggct acaggtctgagttgtgagga ccgtgtaagcagctcttggt ctgttgggt
         Probe # 31           Probe # 32           Probe # 3

cggttcgcttcttacaccaacgacatagccctcgccaagaacgtcgcagcattcatcatc
gccaagcgaag atgtggttgctgtatcggga      cttgcagcgtcgtaagtagt
3           Probe # 34                Probe # 35

ctcaacgacggaatccccatcatctacgccggccaagaacagcactacgccggcggaaac
      ctgccttaggggtagtagat
      Probe # 36

gaccccgcgaaccgcgaagcaacctggctctcgggctacccgaccgacagcgagctgtac

aagttaattgcctccgcgaacgcaatccggaactatgccattagcaaagatacaggattc
ttcaattaacggaggcgctt  gttaggccttgatacggtaa cgtttctatgtcctaag
Probe # 37            Probe # 38           Probe # 39
gtgacctacaagaactggcccatctacaaagacgacacaacgatcgccatgcgcaagggc
cac  gatgttcttgaccgggtaga gtttctgctgtgttgctagc
     Probe # 40           Probe # 41

acagatgggtcgcagatcgtgactatcttgtccaacaagggtgcttcgggtgattcgtat
        cagcgtctagcactgataga             acgaagcccactaagcata
        Probe # 42                       Probe # 43

accctctccttgagtggtgcgggttacacagccggccagcaattgacggaggtcattggc
t                                     cgttaactgcctccagtaac
                                      Probe # 44

tgcacgaccgtgacggttggttcggatggaaatgtgcctgttcctatggcaggtgggcta
                           cctttacacggacaaggata           at
                           Probe # 45                     Pr

cctagggtattgtatccgactgagaagttggcaggtagcaagatctgtagtagctcg
ggatcccataacataggc              tccatcgttctagacatcat
obe # 46                        Probe # 47
```

Supplementary Figure S1. smFISH probe for *amyB.* In 1,497 b of *amyB* sequence, 47 regions (indicated as Probe #) of 18-22 nt were selected for the smFISH probe, each attached with FAM fluorescence molecule.

```
atggaagaggaagttgctgctctcgtcattgacaatggttcgggtatgtgcaaggccggt
         cttcaacgacgagagcagta   gttaccaagcccatacacgt
         Probe # 1              Probe # 2

ttcgccggtgacgatgcccccgtgctgtcttcccctccattgtcggtcgtccccgtcat
                      cacgacagaaggggaggtaa     agcaggggcagta
                      Probe # 3                Probe # 4

catggtatcatgattggtatgggtcagaaggactcttatgtcggtgatgaggcacagtcc
gtaccat  tactaaccatacccagtctt   gagaatacagccactactcc    cagg
         Probe # 5              Probe # 6                Prob

aagcgtggtatcctcaccctcagatatcctatcgagcacggtgtcgtcacaaactgggat
ttcgcaccataggagt    gtctataggatagctcgtgc     cagtgtttgacccta
e # 7               Probe # 8                Probe # 9

gacatggagaagatttggcatcacacattctacaacgagctccgtgttgctcccgaggag
ctgta  tcttctaaaccgtagtgtgt  gatgttgctcgaggcacaac
       Probe # 10            Probe # 11

cacccggttctcctgacggaagcccctatcaaccctaagtccaaccgtgagaagatgacc
                       gggatagttgggattcaggt   cactcttctactgg
                       Probe # 12             Probe # 13

cagattgtcttcgagaccttcaacgccccgccttctacgtctccatccaggccgtcctg
gtctaa  gaagctctggaagttgcggg  cggaagatgcagaggtaggt    caggac
        Probe # 14            Probe # 15              Probe

tcactgtatgcctccggtcgtaccaccggtatcgttctggattctggtgacggtgtcacc
agtgacatacggag  cagcatggtggccatagcaa   ctaagaccactgccacagtg
# 16            Probe # 17             Probe # 18
cacgttgtccccatctacgagggtttcgccctcccccacgccatctcccgtgttgacatg
 tgcaacaggggtagatgctc                  cggtagagggcacaactgta
 Probe # 19                            Probe # 20

gctggccgtgatttgaccgattacctcatgaagatcctggctgagcgcggttacactttc
 gaccggcactaaactggcta  ggagtacttctaggaccgac  gcgccaatgtgaaag
 Probe # 21            Probe # 22            Probe # 23

tccaccaccgctgagcgtgaaattgtccgtgacatcaaggagaagctttgctacgtcgcc
aggtg  ggcgactcgcactttaacag  actgtagttcctcttcgaaa  atgcagcgg
       Probe # 24            Probe # 25            Probe # 2
ctcgacttcgagcaggagattcagaccgcttctcagagctccagcctcgagaagtcctat
gagctgaagct     tctaagtctggcgaagagtc     gtcggagctcttcaggata
6               Probe # 27               Probe # 28

gagcttcctgatggccaggtcatcaccatcggtaacgagcgtttccgtgctcctgaggct
c  gaaggactaccggtccagta  ggtagccattgctcgcaaag   cgaggactccga
   Probe # 29            Probe # 30             Probe # 31

ctcttccagcctagcgttctgggtctggaaagcggtggtatccacgttaccaccttcaac
gagaaggt  gatcgcaagacccagacctt      ccataggtgcaatggtggaa  tg
          Probe # 32                Probe # 33            Pr

tccatcatgaagtgtgatgttgatgtccgtaaggatctctacggtaacatcgtcatgtct
aggtagtacttcacacta  actacaggcattcctagaga  ccattgtagcagtacaga
obe # 34            Probe # 35            Probe # 36

ggtggtactaccatgtaccctggcatctccgatcgtatgcagaaggaaatcaccgccctt
cc     gatggtacatgggaccgtag   ctagcatacgtcttccttta       gaa
       Probe # 37             Probe # 38                 Pro
gccccctcgtccatgaaggtcaagatcattgctcctcctgagcgtaaatactccgtctgg
cgggggagcaggtactt   gttctagtaacgaggaggac  gcatttatgaggcagacc
be # 39             Probe # 40            Probe # 41

atcggtggttccatcttggcttccctgtccaccttccagcagatgtggatctccaagcag
ta  caccaaggtagaaccgaagg  caggtggaaggtcgtctaca  tagaggttcgtc
    Probe # 42            Probe # 43            Probe # 44

gagtacgacgagagcggtccttcgatcgttcaccgcaagtgcttctaa
ctcatgct          ggaagctagcaagtggcgtt
                  Probe # 45
```

Supplementary Figure S2. smFISH probe for *actA.* In 1,128 b of *actA* sequence, 45 regions (indicated as Probe #) of 18-22 nt were selected for the smFISH probe, each attached with CAL Fluor Red 610 fluorescence molecule.

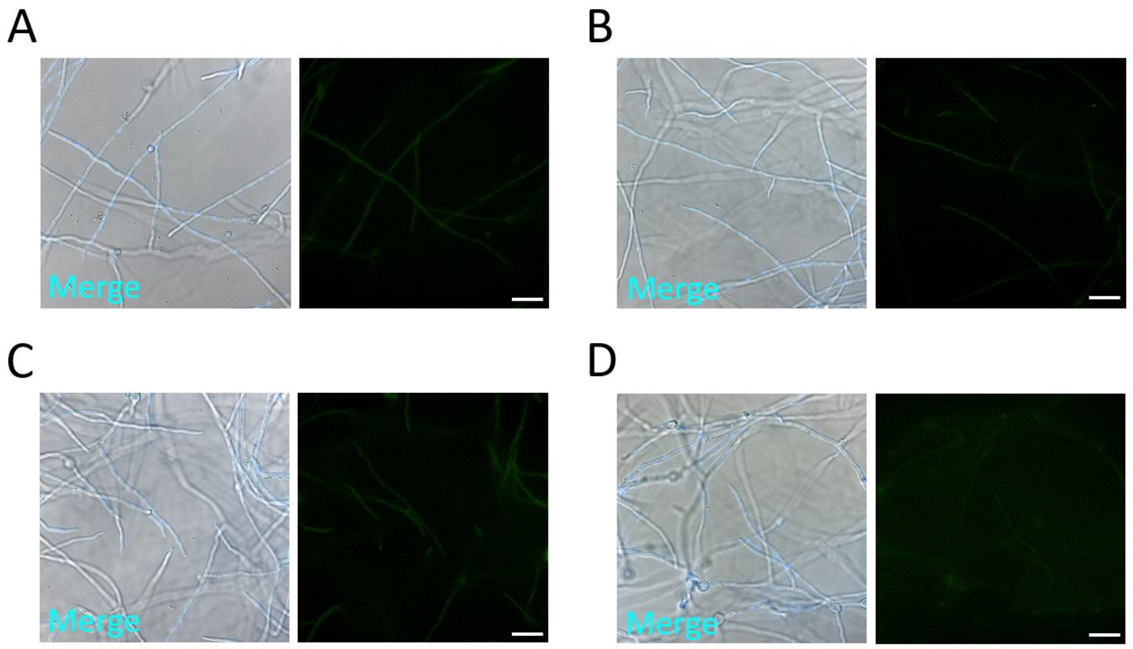

Supplementary Figure S3. smFISH procedures were performed without the *amyB* probe in cultures of CD before shifting (**A**) and after shifting to CDmal, at 30 min (**B**), 60 min (**C**) and 90 min (**D**). Note that almost no fluorescent signals associated with *amyB* were observed (each right panel). Scale bars, 20 µm.

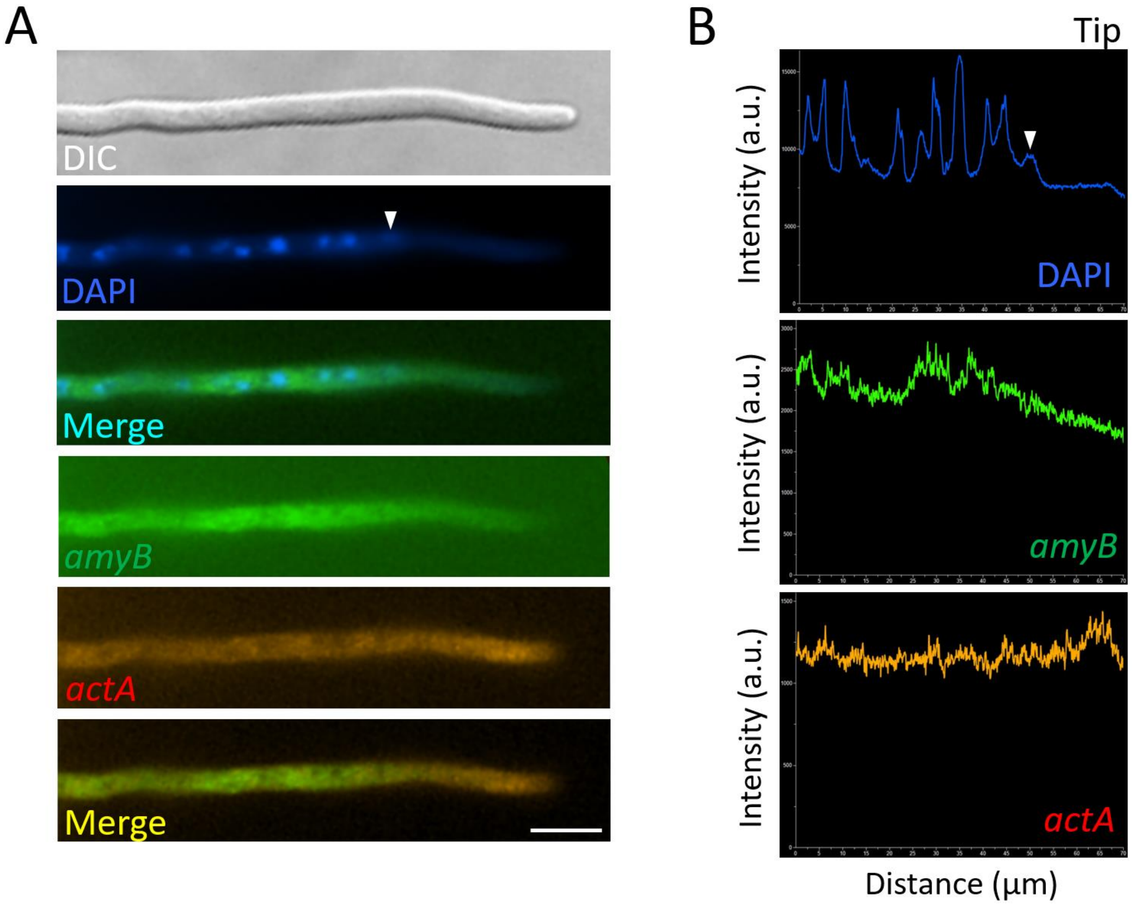

Supplementary Figure S4. Localization of *amyB* and *actA* mRNAs in an *A. oryzae* hypha cultured in complete medium containing maltose. (**A**) smFISH was performed with *amyB* and *actA* probes added to an overnight culture in MPY. The white arrowhead indicates the nucleus located closed to the apex. (**B**) Line scan analyses of DAPI, *amyB* and *actA* mRNAs fluorescent signals were conducted through the hypha of (**A**). The white arrowhead indicates the nucleus located closed to the apex. Note that the fluorescent signals of *amyB* mRNAs are not seen in the apical region, whereas those of *actA* mRNAs appeared more intense in the apical region. Scale bar, 10 µm.
